# Bioflocculant production from untreated corn stover using *Cellulosimicrobium cellulans* L804 isolate and its application to harvesting microalgae

**DOI:** 10.1186/s13068-015-0354-4

**Published:** 2015-10-20

**Authors:** Weijie Liu, Chenchu Zhao, Jihong Jiang, Qian Lu, Yan Hao, Liang Wang, Cong Liu

**Affiliations:** School of Life Science, The Key Laboratory of Biotechnology for Medicinal Plant of Jiangsu Province, Jiangsu Normal University, No.101, Shanghai Road, Tongshan new District, Xuzhou, 221116 Jiangsu China

**Keywords:** Bioflocculant, *Cellulosimicrobium cellulans*, Corn stover, Microalgae harvest, Biofuels

## Abstract

**Background:**

Microalgae are widely studied for biofuel production. Nevertheless, harvesting step of biomass is still a critical challenge. Bioflocculants have been applied in numerous applications including the low-cost harvest of microalgae. A major bottleneck for commercial application of bioflocculant is its high production cost. Lignocellulosic substrates are abundantly available. Hence, the hydrolyzates of rice stover and corn stover have been used as carbon source to produce the bioflocculant in previous studies. However, the hydrolyzates of biomass required the neutralization of pH before the downstream fermentation processes, and the toxic by-products produced during hydrolysis process inhibited the microbial activities in the subsequent fermentation processes and contaminated the bioflocculant product. Therefore, strains that can secrete plant cell-wall-degrading enzymes and simultaneously produce bioflocculants through directly degrading the lignocellulosic biomasses are of academic and practical interests.

**Results:**

A lignocellulose-degrading strain *Cellulosimicrobium cellulans* L804 was isolated in this study, which can produce the bioflocculant MBF-L804 using untreated biomasses, such as corn stover, corn cob, potato residues, and peanut shell. The effects of culture conditions including initial pH, carbon source, and nitrogen source on MBF-L804 production were analyzed. The results showed that over 80 % flocculating activity was achieved when the corn stover, corn cob, potato residues, and peanut shell were used as carbon sources and 4.75 g/L of MBF-L804 was achieved under the optimized condition: 20 g/L dry corn stover as carbon source, 3 g/L yeast extract as nitrogen source, pH 8.2. The bioflocculant MBF-L804 contained 68.6 % polysaccharides and 28.0 % proteins. The Gel permeation chromatography analysis indicated that the approximate molecular weight (MW) of MBF-L804 was 229 kDa. The feasibility of harvesting microalgae *Chlamydomonas reinhardtii* and *Chlorella minutissima* using MBF-L804 was evaluated. The highest flocculating efficiencies for *C. reinhardtii* and *C. minutissima* were 99.04 and 93.83 %, respectively.

**Conclusions:**

This study shows for the first time that *C. cellulans* L804 can directly convert corn stover, corn cob, potato residues and peanut shell into the bioflocculants, which can be used to effectively harvest microalgae.

**Electronic supplementary material:**

The online version of this article (doi:10.1186/s13068-015-0354-4) contains supplementary material, which is available to authorized users.

## Background

In recent years, microalgae have received increasing attention for the production of biofuels and various value-added products [1–5]. However, economic production of these products is hampered by high cost in harvesting and dewatering of biomass, which can account for 20–30 % of total cost [3, 6–8]. Existing technologies used for harvesting microalgal cells include centrifugation, filtration, flotation, gravity sedimentation, and flocculation, either used individually or in combination [1, 2, 6]. Centrifugation can harvest more than 90 % of the microalgae, but requires extra energy input [9–12]. Filtration is effective only for the large multicellular microalgae, and is not economical due to the low efficiency and the frequent filter replacement [12, 13]. Coagulation/flocculation followed by gravity sedimentation or flotation is a relative inexpensive approach for microalgae harvest [14–19]. Co-cultivation of some fungal strains have been reported to be able to assist algal flocculation [6, 20, 21], however, this method requires long culture time [3], and is not applicable for harvesting all microalgae.

Bioflocculants are extracellular polymeric substances including polysaccharides, proteins and nucleic acids secreted by microorganisms [22–24]. Bioflocculants are advantageous over inorganic flocculants and chemically synthetic flocculants in numerous applications including low-cost harvest of microalgae and wastewater treatment, due to their nontoxic, harmless, and biodegradable properties [3, 23, 25, 26]. The bioflocculants produced by *Solibacillus silvestris* W01 [3], *Paenibacillus* sp. AM49 [7, 16], and *Burkholderia cepacia* [27] have been applied in microalgae harvest. However, a major bottleneck for commercial application of these bioflocculants is the high production cost [28, 29]. Current studies commonly focused on isolating high production strains, optimizing fermentation conditions and seeking for low-cost substrates [30–32]. Activity sludge was applied as raw material to produce bioflocculants [33–36], and various wastewaters were used as cheap carbon source to reduce the production cost, such as potato starch wastewater [37–39], palm oil mill effluent [40, 41], dairy wastewater [31], chromotropic acid wastewater [42], and brewery wastewater [43]. Agricultural wastes, such as rice stover and corn stover, are abundantly available and rich in lignocelluloses, hydrolyzates of which have been applied as carbon source to produce bioflocculants [29, 44]. However, the hydrolyzate of biomass requires the neutralization of pH before the downstream fermentation processes [29, 44, 45], and the hydrolyzates of biomass always contain toxic by-products, such as phenolic compounds and furan derivatives, which inhibit the microbial activities in the subsequent fermentation processes [46–48], and are difficult to remove from the bioflocculant product. In some cases, the algae or algal residues are used to produce other valuable products, such as proteins and carbohydrates [2]. Thus, the contamination of bioflocculants by these toxic by-products will limit their application. Therefore, strains that can secrete lignocellulolytic enzymes and simultaneously produce bioflocculants through directly degrading lignocellulosic biomasses are of academic and practical interests.

In this study, a lignocellulose-degrading strain *Cellulosimicrobium cellulans* L804, which can produce bioflocculant MBF-L804 through degrading lignocellulosic biomasses directly, was isolated from corn farmland soil. Subsequently, the optimal fermentation conditions of strain L804 were investigated, and the feasibility of harvesting two microalage *Chlamydomonas reinhardtii* and *Chlorella minutissima* using MBF-L804 was evaluated. The results showed that *C. cellulans* L804 can convert untreated corn stover, corn cob, potato residues, and peanut shell into bioflocculants, which exhibited high flocculating activities in microalgae harvest. Therefore, this study provides a novel way to produce bioflocculant, and achieve the resourceful utilization of abundantly available lignocellulosic biomass, which can promote the industrial application of bioflocculant in microalgae harvest.

## Results and discussion

### Isolation and identification of bioflocculant-producing strains

**Fig. 1:**
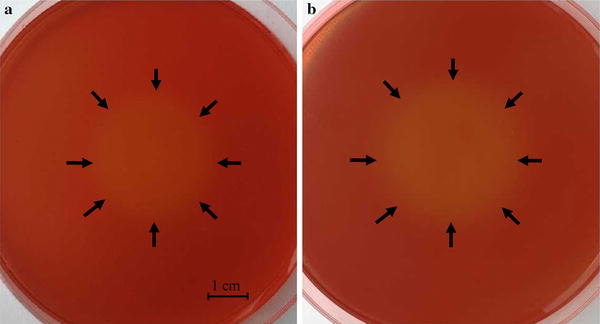
Cellulase (**a**) and xylanase (**b**) of *C. cellulans* L804 analyzed using the medium containing CMC or Xylan. Images were taken at the culture time of 72 h. The *bar* indicates 1 cm

Twelve cellulase-producing and xylanase-producing strains were isolated from corn farmland soil samples. Among them, one strain, named L804, was identified as a bioflocculant-producing strain with high flocculating activity. Strain L804 was Gram positive, rod shaped, and aerobic strain. The colony of L804 was circular and moist. The 16S rRNA of strain L804 was sequenced after PCR amplification and deposited into GenBank database (accession number: KT280277). A total of 1400 bp of 16S rRNA was determined and compared with the sequences of the GenBank database. The highest level of 16S rRNA sequence similarity to *Cellulosimicrobium cellulans* was 99 %. Therefore, strain L804 and its bioflocculant product were named *C. cellulans* L804 and MBF-L804, respectively. In previous studies, *C. cellulans* has been reported to produce an array of plant cell-wall-degrading enzymes, such as endo-β-1,3-glucanases, proteases, and mannanases [49]. *C. cellulans* has also been reported to secrete the lignocellulolytic enzymes, such as cellulase and xylanase, and thus has the capacity for degrading lignocellulosic biomass [44, 50, 51]. In this study, *C. cellulans* L804 was also found to be able to secrete cellulase and xylanase (Fig. 1). More importantly, it was observed for the first time that *C. cellulans* produces bioflocculant. Therefore, strain L804 has potential to produce bioflocculant by degrading the lignocellulosic biomass directly.

In previous studies, the hydrolyzates of some lignocellulosic biomasses, such as rice stover [44] and corn stover [29], were used as carbon source to produce the bioflocculants. However, these biomasses were hydrolyzed under high-temperature and strong acidic conditions (121 °C and 1.7 % sulfuric acid) [29, 44]. After acidic hydrolysis, a neutralization of pH is necessary for the downstream fermentation processes [29, 44, 45], and the acidic hydrolysis process always produces toxic by-products, such as phenolic compounds, furan derivatives, and carboxylic acids, which are major fermentation inhibitors for the activities of bioflocculant-producing strain [52], and are difficult to remove in the extraction process, and thus contaminate the bioflocculant product. In this study, *C. cellulans* L804 was found to be able to utilize untreated lignocellulosic biomasses as carbon sources to produce bioflocculant, which can achieve the resourceful utilization of lignocellulosic biomass.

### MBF-L804 production in the medium with different initial pH

The initial pH of the medium was adjusted using Na_2_CO_3_ and HCl solution. The effects of pH variation in the range of 6.0-10.5 on the cell growth and MBF-L804 production were analyzed. As shown in Table 1, *C. cellulans* L804 produced bioflocculant MBF-L804 in the initial pH range of 7.4–9.8, and the final pH range was 8.5–9.2. Therefore, *C. cellulans* L804 is an alkali-resistant strain. Previous studies reported that weak alkaline treatment opens up the cell wall of lignocellulosic materials, leading to an increase in internal surface area, a decrease in the degree of polymerization, and a decrease in crystallinity; and the separation of structural linkages between lignin and carbohydrates [45]. Therefore, weak alkaline culture condition can improve the degradation efficiency of lignocellulosic biomass, and the highest flocculating activity of 90.88 % was achieved at initial pH 8.2 (Na_2_CO_3_ concentration was 0.4 g/L). Therefore, 0.4 g/L Na_2_CO_3_ was selected to adjust initial pH of the fermentation medium in the following experiments.

**Table 1:** Effects of initial pH on cell growth and flocculating activity

Initial pH	Final pH	Cell growth (OD_600_)	Flocculating activity^a^ (%)
6.0	7.78 ± 0.15	1.61 ± 0.10	3.82 ± 0.36
6.9	8.03 ± 0.14	1.76 ± 0.02	61.27 ± 4.81
7.4	8.52 ± 0.16	1.04 ± 0.21	88.15 ± 4.55
8.2	9.07 ± 0.03	1.00 ± 0.08	90.88 ± 4.87
9.2	9.14 ± 0.06	0.88 ± 0.02	88.30 ± 4.41
9.8	9.23 ± 0.02	1.20 ± 0.04	83.73 ± 2.56
10.2	9.21 ± 0.02	1.30 ± 0.05	54.50 ± 0.71
10.5	10.18 ± 0.10	0.02 ± 0.01	31.95 ± 6.49

±, indicates standard deviation of at least three replicates

^a^Flocculating activity was determined using Kaolin clay as solid phase

### Effects of nitrogen sources on MBF-L804 production

**Fig. 2:**
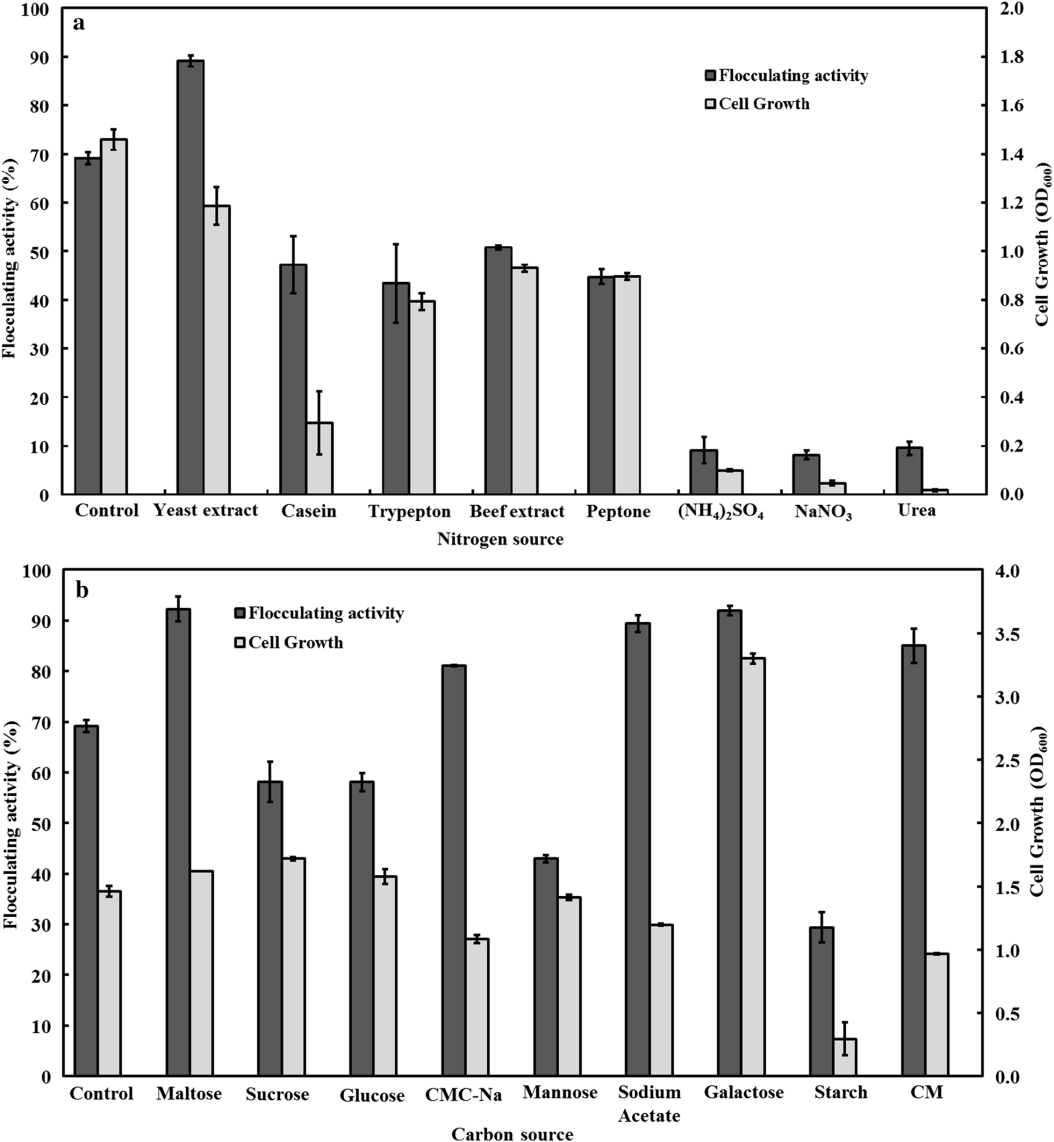
Effects of nitrogen sources (**a**) and carbon sources (**b**) on flocculating activity and cell growth. The control sample indicated that strain L804 cultured in FSS medium. 100 μL of fermentation broth of 48 h was used for flocculating activity assay

The effects of nitrogen sources on MBF-L804 production were investigated. Figure 2a shows the effects of different nitrogen sources on MBF-L804 production. Among the nitrogen sources investigated, ammonium sulfate, sodium nitrate, and urea resulted in poor cell growth and flocculating activity. Comparatively, yeast extract, casein, trypepton, beef extract, and peptone were significantly better sources for MBF-L804 production. Yeast extract was selected as the optimal nitrogen source in the following experiments because it was favorable for the MBF-L804 production.

### Effects of carbon sources on MBF-L804 production

The effects of various carbon sources on MBF-L804 production were then studied when yeast extract was used as nitrogen source. As shown in Fig. 2b, flocculating activity over 80 % was achieved when maltose, galactose, sodium acetate, carboxymethyl cellulose (CMC), and microcrystalline cellulose (CM) were used as carbon sources. More exciting it was to find that strain L804 is able to produce bioflocculant using CMC and CM as carbon sources, confirming that strain L804 has the potential in conversion of lignocellulosic materials into bioflocculants.

### Selection of lignocellulosic biomass to produce bioflocculant

**Fig. 3:**
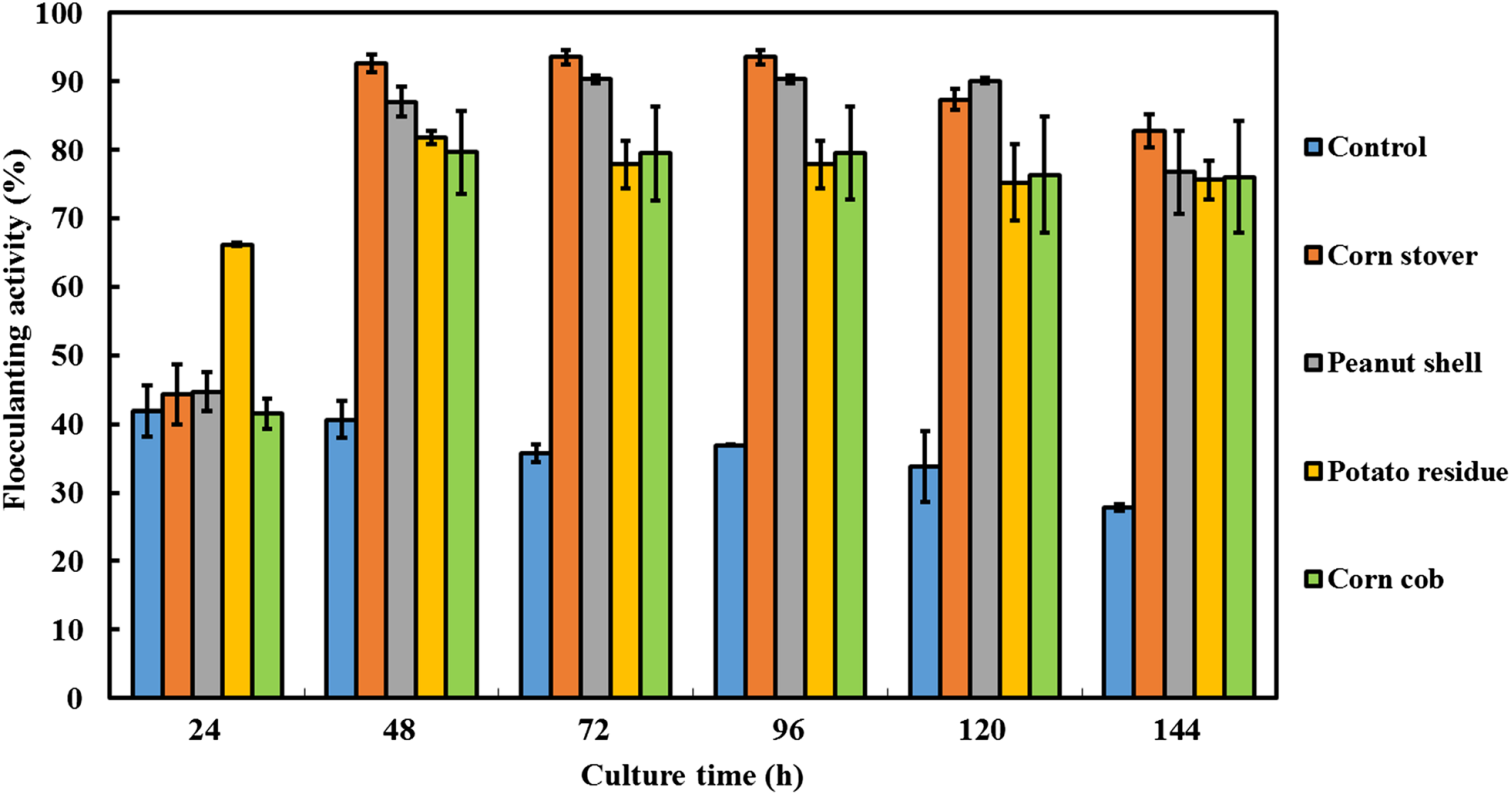
Production of MBF-L804 using different lignocellulosic biomasses as carbon source. Strain L804 was cultured in the mediums (added with 3 g/L yeast extract as nitrogen source and with different biomasses as carbon sources) and the control medium (added with 3 g/L yeast extract, but without added biomasses). 100 μL of fermentation broth of 48 h was taken for flocculating activity assay

Seven kinds of lignocellulosic biomasses, including corn stover, corn cob, rice hull, potato residues, wheat bran, wheat straw, and peanut shell, were used as carbon sources of the fermentation medium. The results showed that over 80 % flocculating activities were achieved when the corn stover, corn cob, potato residues, and peanut shell were used as carbon sources, which were much better than for rice hull, wheat bran, and wheat straw (data not shown). Then, the flocculating efficiencies of the broth-containing corn stover, corn cob, potato residues, and peanut shell were compared at different time intervals. As shown in Fig. 3, the flocculating activities of broth with four kinds of lignocellulosic biomasses were much higher than those of the control without added biomasses, and the highest flocculating activity was achieved when corn stover was used as carbon source. Flocculating efficiency over 90 % was observed after the culture had been grown for 48 h. Therefore, the corn stover was selected as optimal carbon source, and the highest titer of MBF-L804 achieved at the culture time of 48 h was 4.75 g/L, which is much higher than 0.13 g/L of the control broth without added biomasses. After 48 h of culture, the titer decreased gradually with the increase of culture time. Therefore, the culture time of 48 h was selected to extract MBF-L804 in the following experiments. In addition, 1.2 g/L soluble sugar was detected in the medium containing 20 g/L corn stover before inoculating with strain L804. These soluble sugars can promote the initial growth of L804 cells and the secretion of lignocellulolytic enzymes, which further degrade the lignocellulosic biomasses. Soluble sugar of 1.2 g/L was much lower than the titers of bioflocculant MBF-L804 4.75 g/L, suggested that bioflocculant MBF-L804 was mainly produced from the degradation of lignocellulosic biomasses. The bioflocculant titer of 4.75 g/L obtained in this study is much higher than the reported 2.4 g/L bioflocculant secreted by *Rhodococcus erythropolis* in the medium using the hydrolyzates of rice stover as carbon source [44], but is lower than 6 g/L bioflocculant produced by *Ochrobactrum cicero* W2 using the hydrolyzates of corn stover [53]. Although *O. cicero* W2 obtained a higher titer, the acidic hydrolysis of corn stover was under high-temperature and strong acidic conditions (121 °C and 1.7 % sulfuric acid), and the acidic hydrolyzates required the neutralization of pH before the downstream fermentation processes, and the toxic by-products produced during the acidic hydrolysis process contaminated the bioflocculant product.

### Time curves of pH, flocculating activity, cellulase, and xylanase

**Fig. 4:**
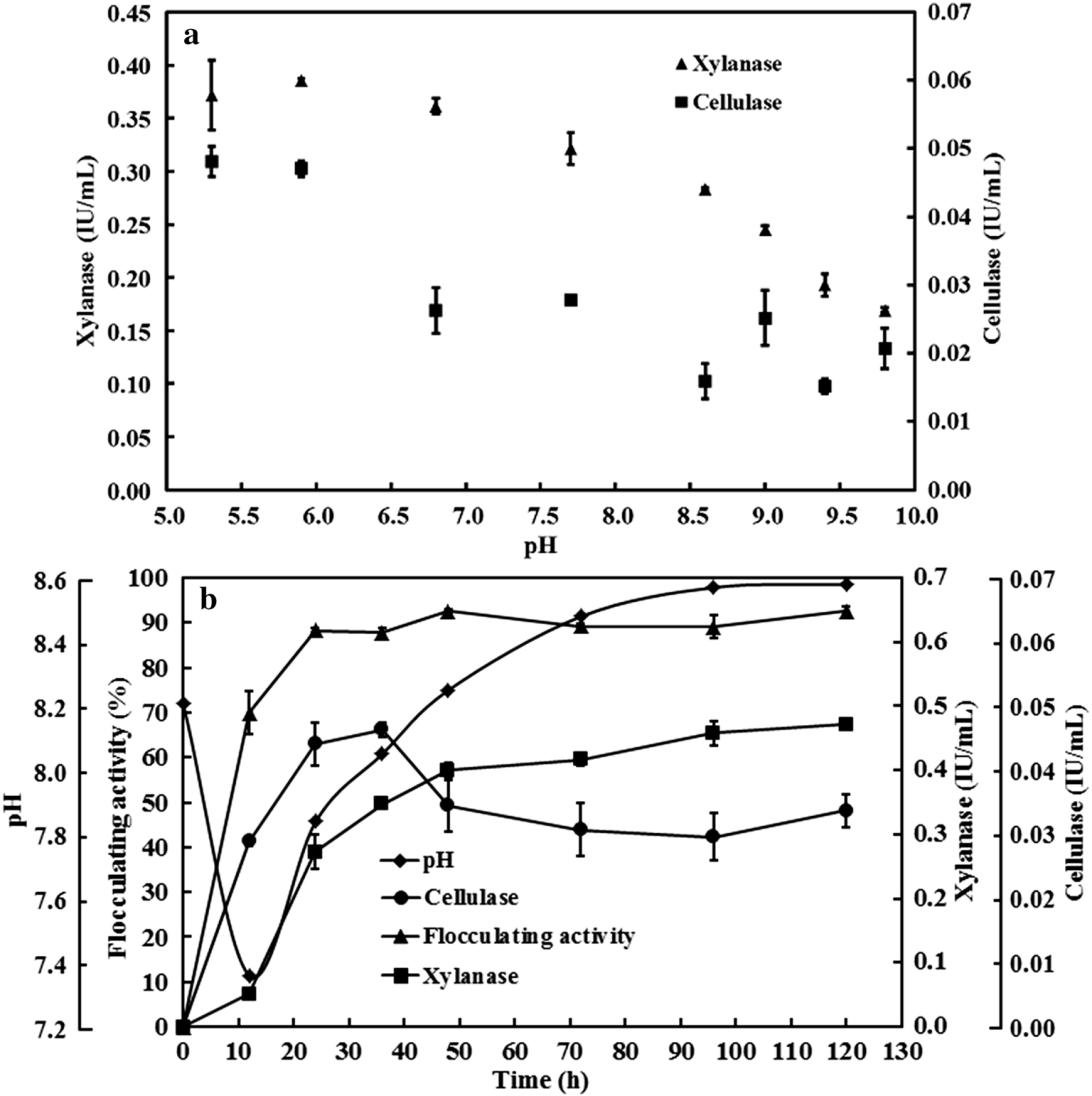
Effects of pH on the activities of cellulase and xylanase produced by *C. cellulans* L804 (**a**) and variation curves of pH, flocculating activity, cellulase, and xylanase during cell growth in fermentation medium with corn stover as carbon source (**b**). *Error bars* indicate standard deviation of at least three replicates

The effects of pH on the activities of xylanase and cellulase present in the supernatant were determined. Figure 4a showed that the optimal pH for these two hydrolytic enzymes was around 5.2–6.0, which was similar to a previous report [50]. Higher pH inhibited their activity. As mentioned above, the initial pH 8.2 of fermentation medium was selected because the bioflocculant was not produced under acidic conditions. Although weak alkaline condition was not the optimal pH for xylanase and cellulase, more than 70 % xylanase activity and 50 % cellulase activity relative to their highest activities remained at the alkaline pH.

The time profiles for pH, flocculating activity of fermentation broth, cellulase, and xylanase were analyzed (Fig. 4b). The pH value decreased sharply from 8.2 to 7.4 in the first 12 h. This decrease may be caused by the release of organic acids during the cell growth [50]. After 12-h culture, the pH increases may be due to the utilization of organic acids as carbon source. A similar change trend of pH was observed in a previous study [50]. Time profiles of cellulase and xylanase activities showed that enzymatic activities were not detectable at the beginning of the fermentation, suggesting that the enzyme content in the lignocellulosic biomass was negligible and that the enzymes were produced only by the microorganism. Figure 4b shows sharp increases in the activities of two enzymes in the early stage of incubation: Xylanase was higher than cellulase. Over 0.6 IU/mL of xylanase activity was achieved after 24-h culture. The highest cellulase activity of 0.046 IU/mL was achieved at 36 h, which then decreased with time. No flocculating activity in fermentation broth was observed at the beginning, and the trends observed for enzyme activities were similar to that of the flocculating activity, suggesting that the conversion from corn stover into bioflocculant was dependent on the activity of hydrolytic enzymes produced by *C. cellulans* L804.

### Characterization of the bioflocculant MBF-L804

The components of MBF-L804 were determined. The results showed that the MBF-L804 contained 68.6 % polysaccharides and 28.0 % proteins. Polysaccharides were the major components of MBF-L804. Gel permeation chromatography analysis indicated that the approximate molecular weight (MW) of the purified bioflocculant L804 was 229 kDa. To reveal the functional groups involved in the flocculating activity of MBF-L804, the FTIR spectrum of the MBF-L804 was analyzed (Additional file 1; Figure S1). The results showed that the MBF-L804 displayed a broad peak at around 3300 cm^−1^, indicating the presence of hydroxyl groups, and the spectrum also displayed a stretching band at 1680 cm^−1^ and a weak symmetric stretching band near 1420 cm^−1^, which are indicative of carboxyl groups. The absorption around 1080 cm^−1^ is known to be a characteristic for all sugar derivatives. The FTIR spectrum was consistent with the results of most bioflocculants produced by other organisms [28, 32, 54, 55].

### Flocculating properties of the bioflocculant MBF-L804

**Fig. 5:**
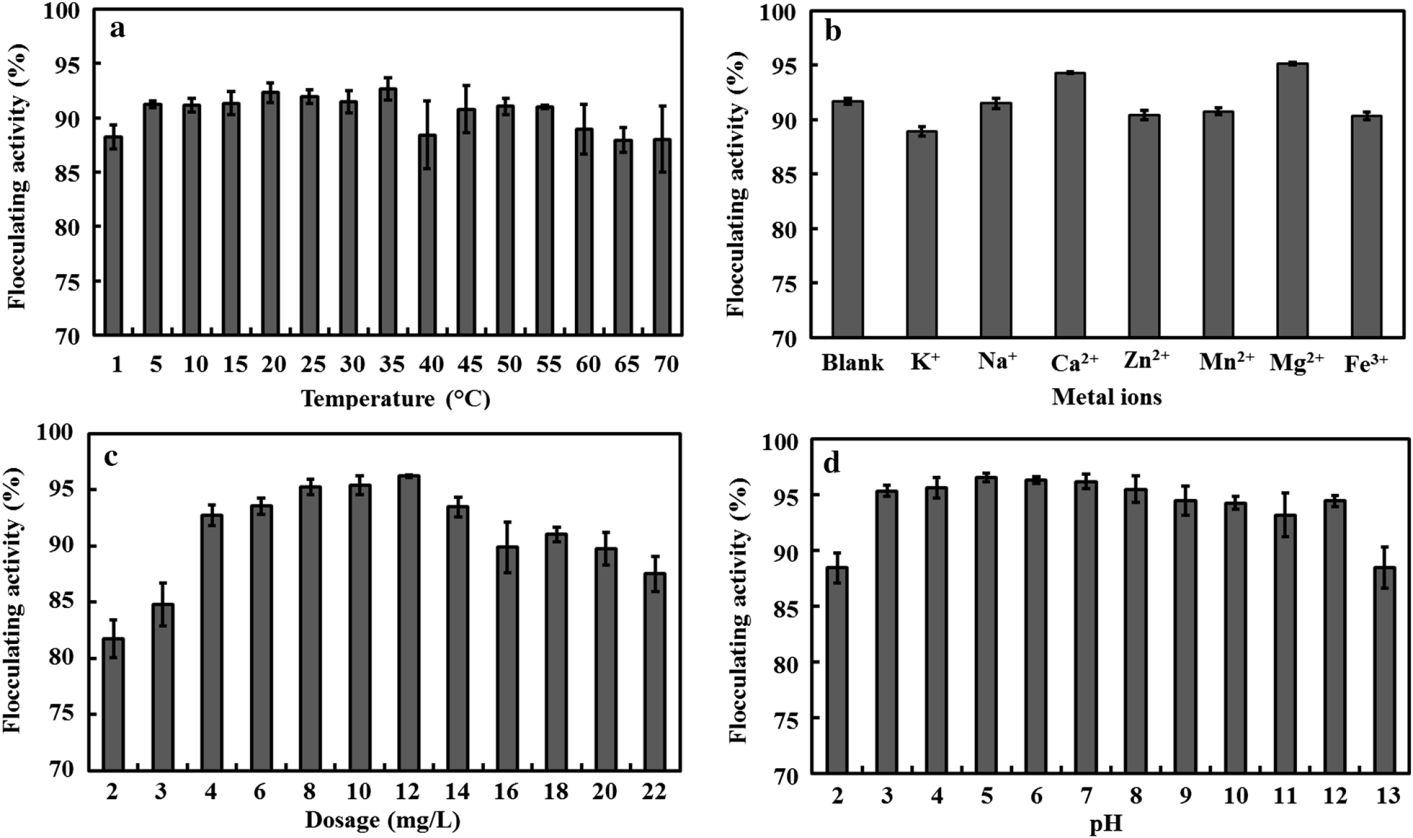
Effects of temperature, metal ion, dosage, and pH on the flocculating activity of MBF-L804

The effects of temperature, metal ion, bioflocculant dosage, and pH on the flocculating activity were evaluated when the Kaolin clay was used as solid phase. The temperature is an important factor influencing the flocculating activity [23]. As shown in Fig. 5a, MBF-L804 showed good heat stability. Over 85 % flocculating activity was achieved at all the tested temperatures, and the highest flocculating activity of 92.68 % was achieved at 35 °C. This could be due to the main components of MBF-L804 being polysaccharides which are more heat stable compared with proteins or nucleic acids [22].

The effects of various metal ions on the flocculating acitivity of MBF-L804 were also investigated (Fig. 5b). It was found that the additions of Ca^2+^ and Mg^2+^ evidently enhanced the flocculating efficiency of MBF-L804. Metal ions are important in the process of flocculation [23]. The bioflocculant generated by *Enterobacter aerogenes* required the presence of Zn^2+^ [56]. The flocculating activity of bioflocculant secreted by *Nannocystis* sp. Nu-2 depended strongly on cations [57]. Ca^2+^ ion can enhance the flocculating efficiency of the bioflocculant produced by *Bacillusagaradhaerens* C9 [28]. Cations stimulate the flocculating activity by neutralizing and stabilizing the residual negative charge of functional groups and by forming the bridges between particles [54]. In this study, it was also found that MBF-L804 shows a good flocculating efficiency of 91.67 % without adding any ion (Blank sample), and Ca^2+^ and Mg^2+^ can further improve the flocculating activity of MBF-L804.

As shown in Fig. 5c, the flocculating activity over 90 % was achieved in the concentration range of 4.0–14.0 mg/L. The solution with lower or higher MBF-L804 concentration showed poor flocculating activity. The bridging phenomenon between particles formed insufficiently when the MBF-L804 dosage was lower than 4 mg/L. When the concentration was higher than 14.0 mg/L, the decrease of flocculating activity could be explained by the repulsion between particles with the same negative charge due to the excessive introduction of charged polysaccharides. The similar relationship between bioflocculant concentration and flocculating activity was observed in other reported extracellular bioflocculants [28, 58]. As shown in Fig. 5d, the flocculating activity of Kaolin suspension was over 90 % in a wide pH range from 3 to 12. The very wide pH and temperature ranges adaptable by MBF-L804 indicated the potential of using it in field applications.

### Application of MBF-L804 in harvesting two microalgae

**Fig. 6:**
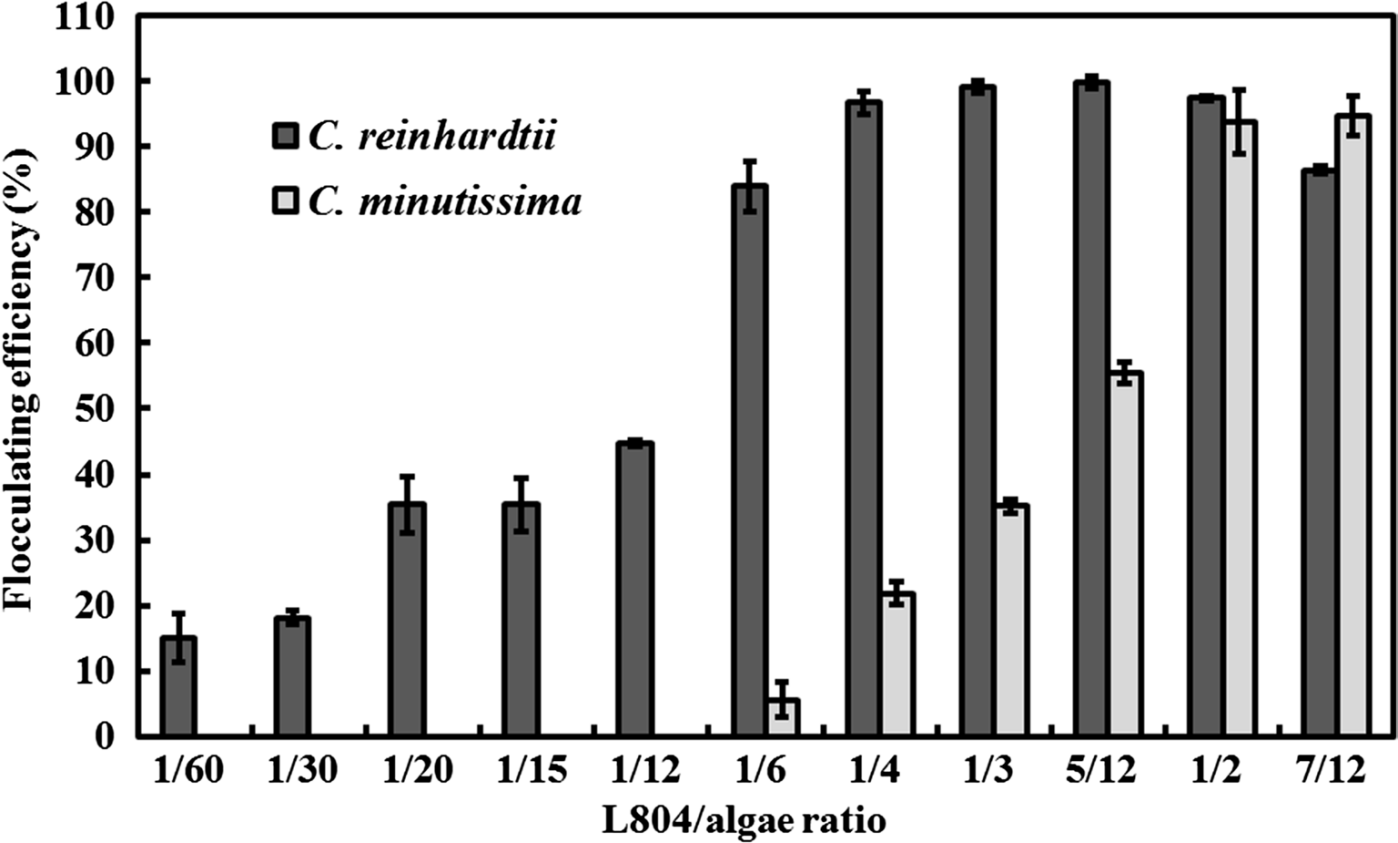
Flocculating efficiencies of *C. reinhardtii* and *C. minutissima* in different volume ratios of L804 fermentation broth/microalgae culture

*Chlamydomonas reinhardtii* and *Chlorella minutissima* are well known microalgae used for biodiesel production research [59, 60]. Compared with traditional methods, such as centrifugation, filtration, and gravity sedimentation, flocculant is a low-cost option to harvest microalgae [9, 18, 61]. Therefore, the feasibility of flocculating *C. reinhardtii* and *C. minutissima* cells using MBF-L804 was investigated in this study. As shown in Fig. 6, the flocculating efficiencies of two microalgae enhanced with the increasing MBF-L804 concentration, and the flocculating efficiency 99.04 % of *C. reinhardtii* was achieved when the culture supernatant of L804 was mixed with *C. reinhardtii* at a ratio of 1/3. The flocculant of *C. minutissima* required more L804 culture supernatant, and flocculating efficiency of 93.83 % was observed when the L804 supernatant was mixed with *C. minutissima* culture in a ratio 1/2, which is significantly lower than the dosage used in previous studies. Wan et al. [3] reported that the bioflocculant produced by *Solibacillus silvestris* W01 can harvest 90 % marine microalga *Nannochloropsis oceanica* DUT01 when the culture supernatant of W01 was mixed with the microalgal culture at a ratio of 3:1. Manheim and Nelson [27] reported that the bioflocculant secreted by *Burkholderia cepacia* could settle microalgae *Scenedesmus* sp. and *Chlorella vulgaris* when algae and bacteria suspensions were mixed in a 2:1 ratio (v/v). In this study, strain L804 can produce bioflocculant by utilizing untreated lignocellulosic biomass as carbon source, which can avoid the capital expenditure intensive pretreatment step, and promote its application in harvesting of microalgae.

## Conclusions

A novel bioflocculant-producing strain *Cellulosimicrobium cellulans* L804 which can secrete lignocellulolytic enzymes was isolated in this study. *C. cellulans* L804 can produce the bioflocculant MBF-L804 by degrading untreated lignocellulosic biomasses. The highest titer of 4.75 g/L MBF-L804 was achieved under the optimal conditions: 20 g/L dry corn stover as carbon source and 3 g/L yeast extract as nitrogen source. The MBF-L804 contained 68.6 % polysaccharides and 28.0 % proteins, and the approximate molecular weight (MW) of MBF-L804 was 229 kDa. In addition, MBF-L804 showed good flocculating efficiencies for *C. reinhardtii* and *C. minutissima* of 99.04 and 93.83 %, respectively.

## Methods

### Isolation of bioflocculant-producing strain

The soil samples were collected from the corn farmland to isolate functional strains which can produce both lignocellulolytic enzymes and bioflocculant. One gram of soil sample was suspended and serially diluted using 0.9 % NaCl solution. The diluted solution was then loaded on the screening medium for isolating cellulase-producing strains and xylanase-producing strains when carboxymethyl cellulose (CMC) and xylan were used, respectively. The composition of the screening medium was as follows: CMC or oat spelt xylan 2 g/L, yeast extract 1 g/L, peptone 1 g/L, casein 1 g/L, K_2_HPO_4_ 1.2 g/L, MgSO_4_·7H_2_O 0.2 g/L, with pH 7.2. After incubation at 37 °C for 48 h, the plates were stained with 0.3 % Congo red dye solution for 10 min, followed by destaining with 1 M NaCl solution for 10 min. The strains forming a clearing zone surrounding the colonies were purified and inoculated into 150-mL Erlenmeyer flasks containing 50 mL flocculating strain screening (FSS) medium (glucose 1 g/L, starch 1 g/L, yeast extract 1 g/L, peptone 1 g/L, casein 1 g/L, K_2_HPO_4_ 1.2 g/L, and MgSO_4_·7H_2_O 0.2 g/L, with initial pH 7.2), and incubated in a shaker at 180 rpm for 48 h at 37 °C. Then, the flocculating rate of each culture broth was determined. Strains with high flocculating activity were selected for further studies.

### Measurement of flocculating activity

The flocculating activities of bioflocculant solution were measured by calculating the flocculating rate according to a previous study [28]. Briefly, Kaolin clay was used as the solid phase, and bioflocculant solution was added into a 5 g/L Kaolin suspension and stirred for 2 min. After settling for 1 min, the absorbance (OD_550_) of the supernatant sample was measured by a spectrophotometer (Unic-7200). A control experiment added with same volume of H_2_O was measured in the same manner. The flocculating rate was calculated according to the following equation: flocculating activity = [A − B]/A × 100 %, where B is the absorbance of the sample at 550 nm, and A is the absorbance of the control at 550 nm.

### Identification of strain L804

The 16S rRNA sequence was analyzed to identify the strain L804. The genomic DNA of L804 was extracted using Genomic DNA Mini Kit (Invitrogen). The 16S rRNA gene fragment of the strain L804 was then amplified by PCR using forward primer (5′-GAG AGT TTG ATC CTG GCT CAG-3′) and reverse primer (5′-CTA CGG CTA CCT TGT TAC GA-3′). The PCR product was purified using a PCR Purification Kit (Tiangen Biotech) and sequenced. The16S rRNA sequence of L804 was deposited into GenBank database (accession number: KT280277) and compared to the sequences available in the GenBank from the National Center for Biotechnology Information (NCBI) Database.

### Effects of initial pH, nitrogen, and carbon sources on MBF-L804 production

The initial pH of the medium was adjusted using Na_2_CO_3_ and HCl solution. The effects of pH variation in the range of 6.0–10.5 on cell growth and MBF-L804 production were evaluated. To analyze the effects of various nitrogen sources and carbon sources on cell growth and MBF-L804 production, the nitrogen sources of FSS medium was replaced by 3 g/L different nitrogen sources, respectively, including yeast extract, casein, trypepton, beef extract, peptone, ammonium sulfate, sodium nitrate, and urea. The carbon sources was changed to 2 g/L different carbon sources, respectively, including maltose, sucrose glucose, CMC-Na, mannose, sodium acetate, galactose, starch, CM when 3 g/L yeast extract was used as nitrogen source.

### Selection of lignocellulosic biomass to produce bioflocculant

To determine which lignocellulosic biomass is favorable for the bioflocculant production, corn stover, corn cob, rice hull, potato residues, wheat bran, wheat straw, and peanut shell, were obtained from Jiangsu province, and crushed into powders and sieved using a 40 mesh sieve. The bioflocculant titers and flocculating activities were compared when 20 g/L of these different dry lignocellulosic biomasses were used as carbon source and 3 g/L yeast extract was used as nitrogen source of the fermentation medium, and the medium (with 3 g/L yeast extract, but without added biomasses) was used as a control to exclude the possibility that the bioflocculant was mainly produced from yeast extract.

### Production, and extraction of the bioflocculant

*C. cellulans* L804 was grown in 50 mL 2 × FSS medium 37 °C overnight. Then, 1 mL seed culture was inoculated into 250-mL flasks containing 100 mL fermentation medium and cultured at 37 °C in a shaker with 200 rpm shaking for 48 h. The composition of the fermentation medium was as follows: untreated dry corn stover 20 g/L, yeast extract 3 g/L, K_2_HPO_4_ 1.2 g/L, MgSO_4_·7H_2_O 0.2 g/L, Na_2_CO_3_ 0.4 g/L, with initial pH 8.2. After 48-h incubation, the fermentation broth was centrifuged at 10,000 rpm at 4 °C for 10 min to remove the cells and corn stover residues. The supernatant was collected for bioflocculant extraction. To extract the bioflocculant MBF-L804, two volumes of cold absolute ethanol were added into the broth to precipitate the bioflocculant. The resulting precipitate was collected by centrifugation at 10,000 rpm, 4 °C for 5 min, washed twice using 75 % ethanol and lyophilized to obtain bioflocculant MBF-L804.

### Enzyme activity assay

To determine the optimal pH for cellulase and xylanase, substrate were dissolved in 0.05 M phosphate buffer (pH 5.2–7.7) and 0.05 M glycine-NaOH buffer (pH 8.6–9.8).

The fermentation broth was collected at different time intervals and centrifuged at 8000 rpm, and the supernatant (enzyme solution) was used for cellulase and xylanase activity assay according to a previous study with slight modification [50]. Cellulase activity was analyzed using carboxymethyl cellulose 1 % (w/v) dissolved in 0.05 M phosphate buffer (pH 5.2) as a substrate. 50 μL enzyme solution was mixed with 150 μL of carboxymethyl cellulose solution. The resulting solution was incubated at 50 °C for 30 min. The reaction was stopped by the addition of 100 μL 1 M NaOH and 150 μL dinitrosalicylic acid (DNS). 50 μL enzyme solution cooked in boiling water for 5 min as control. Then, after adding 550 μL H_2_O, OD_540_ was measured using a spectrophotometer (Unic-7200). One enzyme activity unit was defined as the amount of enzyme that released 1 μM of glucose per minute under the assayed conditions.

Xylanase activity was determined based on the amount of reducing sugars released from 0.5 % (w/v) xylan from oat spelts (Sigma). A 200 μL reaction mixture, containing 50 μL of enzyme solution and 150 μL of a 0.5 % (w/v) suspension of xylan in 0.05 M phosphate buffer (pH 5.9), was incubated at 50 °C for 30 min. The reducing sugars produced were assayed by the DNS method using xylose as standard. 50 μL enzyme was cooked in boiling water for 5 min as control. The OD_540_ was measured using a spectrophotometer (Unic-7200). One enzyme unit was defined as the amount of enzyme that released 1 μM of reducing sugar expressed as xylose equivalents 1 min. All the above measurements were taken in triplicate, and enzyme activities were expressed in IU/mL of supernatant.

### Characteristics of bioflocculant MBF-L804

The total polysaccharides of MBF-L804 were measured using the phenol–sulfuric acid method with glucose as the standard sample [62]. The total protein content was measured by the Bradford method with bovine serum albumin as the standard [63]. The molecular weight was determined by gel permeation chromatography (GPC) using a Hitachi L-6200 system controller [44]. Then, the bioflocculant MBF-L804 was analyzed using a Fourier transform infrared (FTIR) spectroscopy (Bruker Tensor 27, Germany). The spectrum of the sample in the KBr pellet was recorded on the spectrophotometer over a wave-number range of 600–4000 cm^−1^, and processed using the Bruker OPUS software.

### Flocculating properties of MBF-L804

To get the knowledge of flocculating properties of MBF-L804, the effects of temperature, metal ion, bioflocculant dosage, and pH on flocculating activity of Kaolin clay solutions were determined. The temperature of the Kaolin suspension was changed in the range of 1–70 °C. To test the effect of metal ion on the flocculating activity, 1 mL 10 g/L NaCl, KCl, CaCl_2_, ZnCl_2_, MnCl_2_, MgCl_2_, FeCl_3_ solution was introduced into the 60 mL flocculating system. The bioflocculant dosage was varied from 2 to 22 mg L^−1^. The pH of the Kaolin suspension was adjusted using HCl and NaOH in the pH range of 2.1–12.9.

### Culture of two microalgae and its harvest by bioflocculant MBF-L804

*Chlorella minutissima* was cultured in 500-mL Erlenmeyer flask containing 200 mL IM medium [64] which is composed of glucose 17.5 g/L, casein 13 g/L, yeast extract 0.1 g/L, NH_4_Cl 2 g/L, KH_2_PO_4_ 1 g/L, Na_2_HPO_4_ 2 g/L, MgSO_4_·7H_2_O 0.5 g/L, FeSO_4_·7H_2_O 0.01 g/L, CaCl_2_ 0.01 g/L, Al_2_(SO_4_)_3_·18H_2_O 3.58 mg/L, MnCl_2_·4H_2_O 12.98 mg/L, CuSO_4_·5H_2_O 1.83 mg/L, ZnSO_4_·7H_2_O 3.2 mg/L, pH 7.0. *Chlamydomonas reinhardtii* was grown in liquid TAP medium, which is composed of TAP salts (NH_4_Cl 15 g/L, MgSO_4_·7H_2_O 4 g/L, CaCl_2_·2H_2_O 2 g/L) 25 mL/L, Phosphate solution (K_2_HPO_4_ 288 g/L, KH_2_PO_4_ 144 g/L) 0.375 mL/L, Hutner’s trace elements (EDTA disodium salt 50 g/L, ZnSO_4_·7H_2_O 22 g/L, H_3_BO_3_ 11.4 g/L, MnCl_2_·4H_2_O 5.06 g/L, CoCl·6H_2_O 1.61 g/L, CuSO_4_·5H_2_O 1.57 g/L, (NH_4_)_6_Mo_7_O_24_·4H_2_O 1.1 g/L, FeSO_4_·7H_2_O 4.99 g/L, pH 7.0) 1 mL/L, Glacial acetic acid 1 mL/L, and Tris 2.42 g/L. Both two microalgae were cultivated at 23 °C for a 14/10-h light/dark cycle for 1 week. L804 fermentation broth of different volumes was added into 60 mL microalgae culture, and mixed with 0.8 mL 10 % CaCl_2_. After stirring for 5 min and settling for 10 min, the supernatant was removed carefully. Then, the flocculated-microalgae were collected. All the microalgal cells of 60 mL culture were collected by centrifuged at 10,000 rpm 5 min directly as a control. After washing twice with H_2_O, the microalgal samples were lyophilized. The flocculating efficiency was calculated according to the following equation: flocculating efficiency = A/B × 100 %, where A is the dry weight of the flocculated sample, and B is the dry weight of the control sample.

## Additional file

10.1186/s13068-015-0354-4 Fourier transform infrared spectroscopy of bioflocculant MBF-L804.

## Abbreviations

MBF: microbial flocculant
CMC: carboxymethyl cellulose
CM: microcrystalline cellulose
PCR: polymerase chain reaction
DNS: 3, 5-dinitrosalicylic acid
NCBI: National Center for Biotechnology Information
GPC: Gel permeation chromatography
FSS: flocculating strain screening medium
FTIR: Fourier transform infrared spectroscopy
