# Supplementary figures and images for: Bioflocculant production from untreated corn stover using Cellulosimicrobium cellulans L804 isolate and its application to harvesting microalgae
Source: Biotechnol Biofuels. 2015 Oct 20;8:170. doi: 10.1186/s13068-015-0354-4 (PMC4617488; doi:10.1186/s13068-015-0354-4)

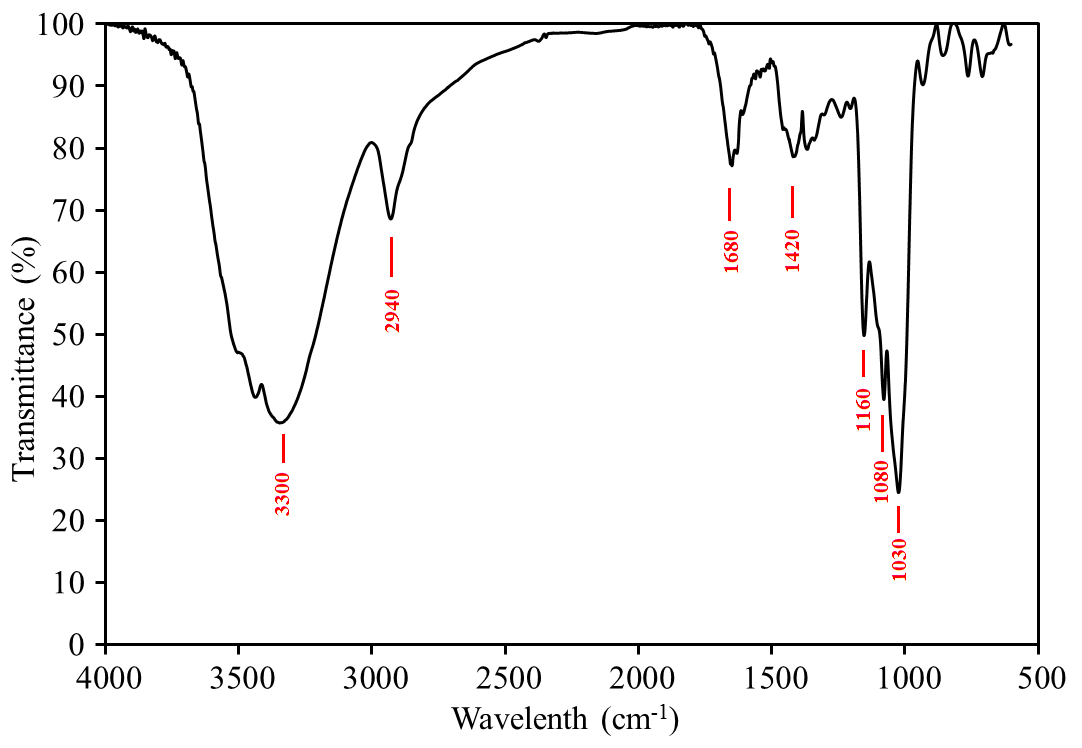

Supplement: Supplementary file 1 — 10.1186/s13068-015-0354-4 Fourier transform infrared spectroscopy of bioflocculant MBF-L804. [file 13068_2015_354_MOESM1_ESM.tif]
